# Equity in Essential Maternal, Newborn, and Child Health Interventions in Northeastern China, 2008 to 2018

**DOI:** 10.3389/fpubh.2020.00212

**Published:** 2020-07-02

**Authors:** Ying Wang, Ran Liao, Xing Lin Feng

**Affiliations:** ^1^Department of Health Policy and Management, School of Public Health, Peking University, Beijing, China; ^2^Department of Immunization Program, Zhejiang Provincial Center for Disease Control and Prevention, Zhejiang, China

**Keywords:** China, maternal newborn and child health, primary care, health system, equity

## Abstract

**Objectives:** We aim to analyze equity in maternal, newborn, and child health (MNCH) interventions in Jilin, a northeastern province of China, 2008–2018.

**Study design:** Cross-sectional study.

**Methods:** We used provincially representative survey data from 2008, 2013, and 2018. We included 18 essential MNCH interventions, analyzed equity, and calculated the composite coverage score. We used logistic and multiple linear regressions to adjust sampling clusters and covariates.

**Results:** Coverage of hospital-based interventions, such as hospital delivery and antenatal B-ultrasound tests, was nearly universal in Jilin province. Cesarean sections persisted at alarmingly high rates (57.6%). Enormous unmet needs and rural–urban inequalities existed for community-based interventions, such as improved drinking water sources (85.4 vs. 97.9%, *p* < 0.01), improved sanitation facilities (52.5 vs. 94.2%, *p* < 0.01), four government-funded antenatal care services (55.8 vs. 84.1%, *p* < 0.01), and at least eight antenatal care sessions (26.8 vs. 46.3%, *p* < 0.05). Compared to rural–urban inequity, individual-level disparities across income and education were either small in scale or statistically insignificant. The inequity in coverage of maternal and newborn care shrank during 2008–2018.

**Conclusions:** Despite its success in reducing mortality, China's unique obstetrician-led safe motherhood strategy may come at the cost of over-medicalization and health inequity. Jilin province's recent efforts to revitalize primary health care show the potential to make a change. An integrated system that links families, communities, and all levels of health care organizations seems to be the most effective and efficient model to offer continuing MNCH care.

## Introduction

Global health initiatives, such as the Millennium Development Goals (MDGs) and the Sustainable Development Goals (SDGs), prioritize maternal, newborn, and child health (MNCH) (1, 2). Consensus was reached to extend health coverage along the MNCH care continuum with the rationale of effectively linking families, communities, and all levels of health care providers as a means to universal coverage (3–5). Focusing on 75 prioritized countries, the *Countdown to 2015* (now *Countdown to 2030*) represents a decade of efforts to monitor the trends of coverage and equity in a series of essential MNCH interventions (6, 7). However, China is the only country that has not yet reported its equity profiles (8, 9).

Notably, China adopted a unique hospital-based birth strategy in the 1990s and successfully achieved MDGs 4 and 5, which required each country to reduce its maternal mortality by three quarters and under-five mortality by two thirds between 1990 and 2015 (10–12). Endorsing the *Law of Maternal and Infant Health* in 1995, the Chinese government established several policies and programs to promote universal hospital births. Hospital deliveries are heavily subsidized with both demand- and supply-side financing mechanisms; community-based traditional birth attendants are abandoned, midwives are marginalized by obstetricians, and certification of birth-attendance services is gradually centralizing from primary care providers to county hospitals and above (10, 13–15). National data in China show that hospital births increased from <50% to almost 100% during 1988 and 2008 (16). The rise in hospital births fully account for the descending trends in maternal mortality and may prevent 48–70% of neonatal deaths in China (10, 17). Although these are positive trends, there are concerns that such a doctor-led approach may usher in over-medicalization and health system fragmentation, which may exacerbate health inequity (12, 18).

Interestingly, China experienced dramatic changes in its health system development. During the 1960s−1980s, China adopted a community-based, people-centered, and de-professionalized health development approach. The World Health Organization (WHO) recognized China's experience in scaling up essential health care coverage, which inspired many principles of primary health care that were promoted in the Alma Ata Declaration (18, 19). However, after the 1980s, China experienced a substantial shift to a laissez-faire paradigm and medicalization, moving the health provision focus from primary health care to disease-centered, highly specialized, and high-cost tertiary care (19, 20). Aware of these problems, the Chinese government has been proposing a new round of health reform since 2009 with health equity as a core issue. This reform aims to reorient the country's health system to primary health care (21–23). One milestone is the *National Essential Public Health Services* program. In this program, the government offers free access to a package of essential health care services and pays particular attention to MNCH (24–26). Previous studies on China's health system reform mainly focused on the general uptake of inpatient or outpatient care, while few reports incorporated the aforementioned contextual changes when specifically analyzing China's safe-motherhood policies (12, 27).

With a land area of 187,000 square kilometers and a population of 27.5 million, Jilin province is located in northeastern China and can be considered a typical province of China in terms of socioeconomic and health system development (please see Appendix 1 for the mapped location and detailed socio-demographic profiles). In this study, we aim to describe the equity of Jilin province in the context of China's health system reform using three waves of provincially representative health survey data from 2008, 2013, and 2018. We attempt to draw lessons for both China and other low- and middle-income countries (LIMCs) on how to strengthen a country's health system effectively and efficiently to extend coverage of essential MNCH interventions.

## Methods

**Figure 1:**
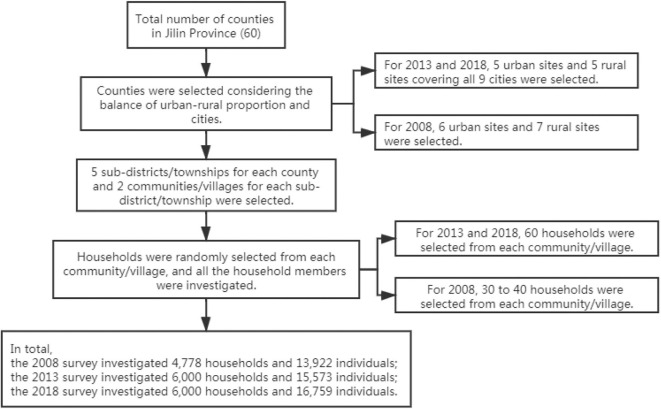
Sampling procedure for NHSS Jilin province.

We used data from three cross-sectional waves of the National Health Service Survey (NHSS) that were conducted in Jilin province, China in 2008, 2013, and 2018. For each wave, expanded provincially representative household samples were obtained using a four-stage, stratified, clustered, random sampling procedure (28). The sampling procedure is shown in Figure 1 (for details of the sampling maps and the population covered, please see Appendix 2). Qualified investigators conducted the household interviews using structured questionnaires. Basic information on households, health status of household members, demands and utilization of health services, and information on maternal and child care was collected. In our study, we used parts of households, mothers with live births in the past 5 years and children under 5 years old.

We included 18 essential MNCH interventions building on the *Countdown*, the latest WHO recommendations on quality antenatal care services that were released in 2016, and China's *National Essential Public Health Services* program (8, 29). The interventions were classified into three domains along the MNCH care continuum according to the target population: water and sanitation, maternal and newborn care, and child care. Within the first domain, we analyzed coverage of improved drinking water sources and sanitation facilities using the *Countdown* definitions (8). Within the maternal and newborn care domain, we analyzed coverage of the following eight interventions: at least one, four, five, and eight sessions of antenatal care; four types of government-funded antenatal services; hospital delivery; cesarean section; and at least one session of postnatal care. We defined a mother as receiving four types of government-funded antenatal services if she received all four types of services that are freely provided in China's *National Essential Public Health Services* program at least once during her antenatal period. The four services are blood testing, blood pressure monitoring, urine testing, and B-ultrasound examination; the former three were analyzed as antenatal care quality in a recent study (30). Note that these services were not included in the 2018 survey. Within the child care domain, we analyzed coverage of growth monitoring services (≥1 check) for all 3 years, early initiation of breastfeeding and exclusive breastfeeding within 6 months in specific years considering comparable definitions of the interventions and the following five vaccinations in 2008 and 2013: one dose of BCG, one dose of measles, three doses of DPT, hepatitis B, and polio vaccines. The intervention variables for households and women had no missing data. For the child-related intervention variables, we excluded missing data. We assumed that the effect was negligible because missing data accounted for only 3.8% of all the survey data although more missing data occurred for children living in rural areas.

**Figure 2:**
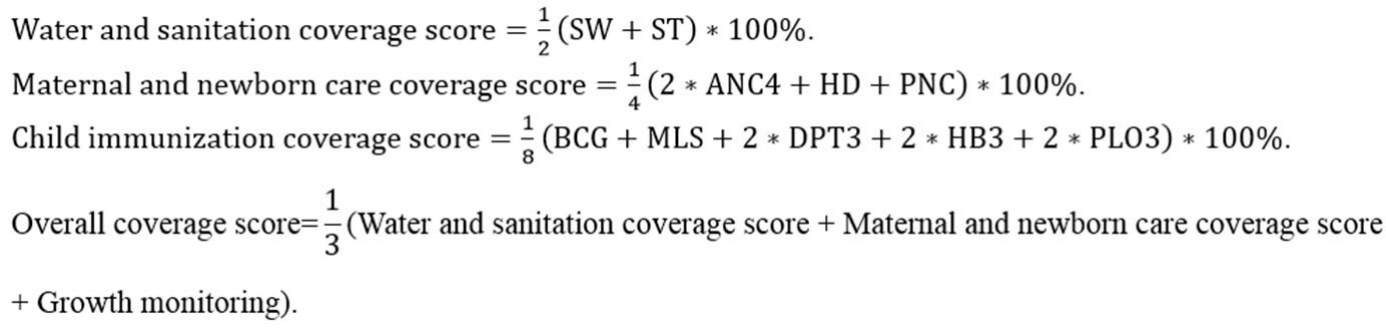
Formulas for building composite coverage scores for essential interventions along the MNCH care continuum. SW, Improved drinking water sources; ST, Improved sanitation facilities; ANC4, At least four antenatal care sessions; HD, Hospital delivery; PNC, At least one postnatal care session; BCG, BCG vaccination; MSL, Measles vaccination; DPT3, DPT vaccination (3 doses); HB3, Hepatitis B vaccination (3 doses); PLO3, Polio vaccination (3 doses). The child immunization coverage score was only available for 2008 and 2013 and was thus not calculated in the overall coverage score.

To better compare the coverage and equity of MNCH interventions, we additionally constructed an overall MNCH care composite coverage score and three specific composite coverage scores for different domains of the MNCH care continuum, adopting the *Countdown* approach: (31, 32) water and sanitation coverage score, maternal and newborn care coverage score, and child immunization coverage score (Figure 2). The three specific composite coverage scores were calculated for each target object. The intervention in the formulas had a value of 1 if used and 0 otherwise. The overall composite coverage score was calculated for the population using the average scores of the specific composite coverage scores in the target population. All the composite scores were transformed to proportions ranging from 0 to 100%. We excluded interventions that were not surveyed in all 3 years from the overall composite coverage score calculation. Cesarean sections were also excluded because there is no consensus on the ideal rates (31), and the actual rates are all higher than 50% in Jilin province. We analyzed correlations between different composite coverage scores and child health outcomes (under-five mortality rates and under-five stunting rates) at the county level to check the validation. The water and sanitation coverage score showed significantly negative correlations with both outcomes. The maternal and newborn care coverage score and the overall composite coverage score showed significantly negative correlations with under-five stunting rates. The child immunization coverage score showed no significant correlations with either outcome, but we retained it because it is still a concern for other LIMCs. For more details on the correlation analysis, please see Appendix 3.

We focused on the coverage equity for different rural–urban settings, educational achievements, and household income groups. We used the rural–urban classification of the National Bureau of Statistics of the People's Republic of China, which defines townships as rural and subdistricts as urban. We grouped households' economic status as poor vs. rich, separated by the median per capita annual income for each survey separately ($730, $1,977, and $2,171 in 2008, 2013, and 2018, respectively). We grouped educational achievements into primary and below and secondary and above.

The survey also collected information on household heads' or mothers' educational achievement, ethnicity, households' distance to the nearest health facility, mothers' and children's health insurance coverage, and other covariates, such as mothers' age at childbirth, parity, and children's age in months and sex. These variables were adjusted in the equity analysis considering the unequal distributions in different groups.

We described the trends of the 18 essential interventions and the composite coverage scores using bar graphs and used chi-square tests to test the differences. We analyzed the equity gaps for these interventions, adopting the dots-plot approach that was developed by the *Countdown* (32). In the *Countdown*, mean coverage rates in each wealth quintile are displayed intuitively by differently colored dots in a line. We used logistic regressions (for each intervention) and multiple linear regressions (for composite coverage scores) adjusting for relevant variables and calculated marginal means for rural–urban settings, poor and rich, and different educational achievement groups. We display the marginal means in each group instead of the unadjusted mean coverage rates used in the *Countdown*. We used multiple linear regressions, accounting for sampling clusters, to analyze the trends and factors associated with the composite coverage scores. These analyses were all implemented in Stata 13.0.

## Results

In total, we analyzed data from 16,778 households, 1,534 mothers, and 1,725 children (Table 1). Household income per capita tripled during 2008–2018 with medians ranging from $730 to $2,171 in Jilin province, China. Almost all of the mothers achieved primary and above education. Social insurance coverage increased from 70% to nearly universal from 2008 to 2018 for mothers, but for children, it was relatively low at 81.6% in 2018. Multiparity increased from below 30% in 2008 and 2013 to 41.8% in 2018, and 85% of the mothers gave birth at the age of 20–34.

**Table 1:** Characteristics of investigated households, mothers, and children, Jilin province, China (2008–2018)^a^.

	**2008**		**2013**		**2018**		***X^**2**^***	***P***
**Total household number**	**4,778**		**6,000**		**6,000**			
**Region**								
Rural	2,658	55.6	3,481	58.0	3,120	52.0	44.45	<0.01
Urban	2,120	44.4	2,519	42.0	2,880	48.0		
**Ethnic group of household head**	**4,759**							
Others	575	12.1	707	11.8	533	8.9	36.92	<0.01
Han	4,184	87.9	5,293	88.2	5,467	91.1		
**Household annual income per capita^b^**					5,997			
Poorer 50%	2,451	51.3	3,141	52.4	3,066	51.1	2.07	0.356
Richer 50%	2,327	48.7	2,859	47.7	2,931	48.9		
**Education of household head**	**4,747**							
Illiterate	310	6.5	284	4.7	275	4.6	54.15	<0.01
Primary	2,995	63.1	3,724	62.1	3,568	59.5		
Secondary and above	1,442	30.4	1,992	33.2	2,157	36.0		
**Time to the nearest health facility**	**4,755**		**5,996**					
>15 min	660	13.9	1,200	20.0	667	11.1	192.84	<0.01
≤15 min	4,095	86.1	4,796	80.0	5,333	88.9		
**Medical insurance coverage of household head**	**4,759**							
No	1,012	21.3	358	6.0	270	4.5	999.36	<0.01
Yes	3,747	78.7	5,642	94.0	5,730	95.5		
**Women aged 15–49 with a live birth in last 5 years**	**562**		**493**		**479**			
**Age when giving birth**								
<20 years old	13	2.3	19	3.9	13	2.7	51.63	<0.01
20–23 years old	156	27.8	134	27.2	55	11.5		
24–34 years old	332	59.1	286	58.0	346	72.2		
≥35 years old	61	10.9	54	11.0	65	13.6		
**Parity**								
Multiparity	167	29.7	124	25.2	200	41.8	32.91	<0.01
Primiparity	395	70.3	369	74.8	279	58.2		
**Non-communicable disease**								
No	547	97.3	475	96.3	426	88.9	39.69	<0.01
Yes	15	2.7	18	3.7	53	11.1		
**Ethnic group**								
Others	49	8.7	50	10.1	41	8.6	0.91	0.634
Han	513	91.3	443	89.9	438	91.4		
**Education**	**560**							
Illiterate	15	2.7	3	0.6	1	0.2	68.05	<0.01
Primary	372	66.4	323	65.5	228	47.6		
Secondary and above	173	30.9	167	33.9	250	52.2		
**Coverage of medical insurance**								
No	170	30.2	49	9.9	30	6.3	130.58	<0.01
Yes	392	69.8	444	90.1	449	93.7		
**Children aged 0–59 months**	**620**		**566**		**539**			
**Month age**								
0–11	138	22.3	89	15.7	90	16.7	19.00	0.02
12–23	129	20.8	125	22.1	104	19.3		
24–35	129	20.8	119	21.0	110	20.4		
36–47	116	18.7	110	19.4	96	17.8		
48–59	108	17.4	123	21.7	139	25.8		
**Sex**								
Female	270	43.5	284	50.2	261	48.4	5.65	0.06
Male	350	56.5	282	49.8	278	51.6		
**Ethnic group**								
Others	67	10.8	74	13.1	65	12.1	1.46	0.48
Han	553	89.2	492	86.9	474	87.9		
**Education of household head**	**611**							
Illiterate	31	5.1	5	0.9	16	3.0	76.20	<0.01
Primary	421	68.9	411	72.6	282	52.3		
Secondary and above	159	26.0	150	26.5	241	44.7		
**Coverage of medical insurance**								
No	337	54.4	127	22.4	99	18.4	209.71	<0.01
Yes	283	45.6	439	77.6	440	81.6		

a *In 2008, there were 23, 19, 31, 19 and 2 missing values for time to the nearest health facility, household head's health insurance coverage, educational achievement, ethnicity, and mother's educational achievement, respectively; in 2013, there were 4 missing values for time to the nearest health facility; and in 2018, there were 3 missing values for household annual income per capita*.

b *Economic status was defined by household annual income per capita, grouped as poor vs. rich, using the median per capita income as the dividing threshold for the 2008, 2013, and 2018 samples separately. The thresholds were $730 in 2008, $1,977 in 2013 and $2,171 in 2018*.

**Figure 3:**
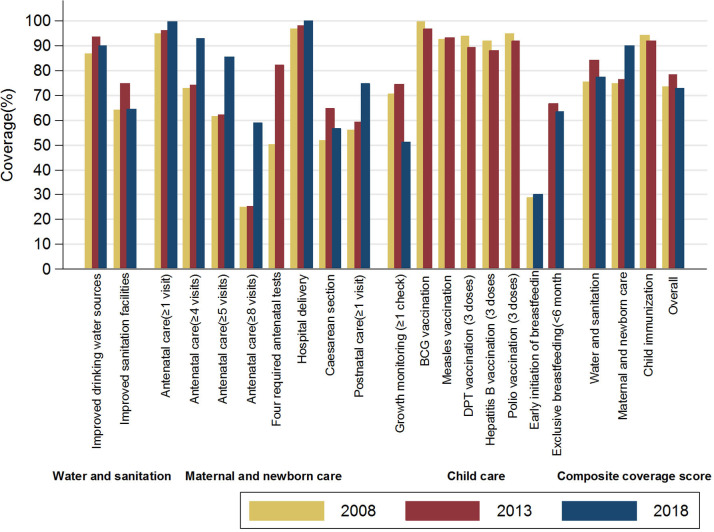
Essential MNCH intervention coverage along the care continuum, Jilin province, China (2008–2018).
Four government-funded antenatal services, defined as whether the mother received all four types of services at least once during her antenatal period. These services are freely provided by China's *National Essential Public Health Services* program and include blood testing, blood pressure monitoring, urine testing, and B-ultrasound examinations.Water and sanitation coverage score is the composite coverage score for improved drinking water sources and improved sanitation facilities.Maternal and newborn care coverage score is the composite coverage score for antenatal care, hospital delivery, and postnatal care.Child immunization coverage score is the composite coverage score for five types of child immunization.Overall coverage score is the composite coverage score for water and sanitation, maternal and newborn care, and child growth monitoring. Child immunization coverage scores were excluded because we lacked data in 2018.

As Figure 3 shows, coverage of hospital delivery and each of the five child vaccinations had already been above 90.0% since 2008. The proportions of mothers who received at least four antenatal care sessions (from 73.0% in 2008 to 92.9% in 2018), at least five antenatal care sessions (from 61.6% in 2008 to 85.4% in 2018), at least eight antenatal care sessions (from 25.1% in 2008 to 58.9% in 2018), and at least one postnatal care session (from 56.0% in 2008 to 74.7% in 2018) were relatively low but increased significantly since 2013 (*p* < 0.01). The proportion of cesarean sections increased from 51.8 to 64.9% during 2008–2013 and decreased to 56.8% in 2018. Falls after a rise were observed for coverage of improved drinking water sources (from 86.7% in 2008 to 93.7% in 2013 and to 90.2% in 2018, *p* < 0.01), improved sanitation facilities (from 64.1% in 2008 to 75.0% in 2013 and to 64.6% in 2018 again, *p* < 0.01), and at least one session of growth monitoring (from 70.6% in 2008 to 74.5% in 2013 and to 51.0% in 2018, *p* < 0.01). Additionally, the four types of government-funded antenatal care services increased significantly from 50.4 to 82.2% during 2008–2013. Coverage of early initiation of breastfeeding (~30%) and exclusive breastfeeding within 6 months (~65%) seemed sluggish during 2008–2018. In sum, the water and sanitation coverage score rose from 75.4% in 2008 to 84.3% in 2013 and then fell back to 77.4% in 2018; the maternal and newborn care coverage score increased from ~75% during 2008–2013 to 90.1% in 2018; and the overall coverage scores were 73.6, 78.4, and 72.8% in 2008, 2013, and 2018, respectively. The child immunization coverage scores were all higher than 90%.

**Figure 4:**
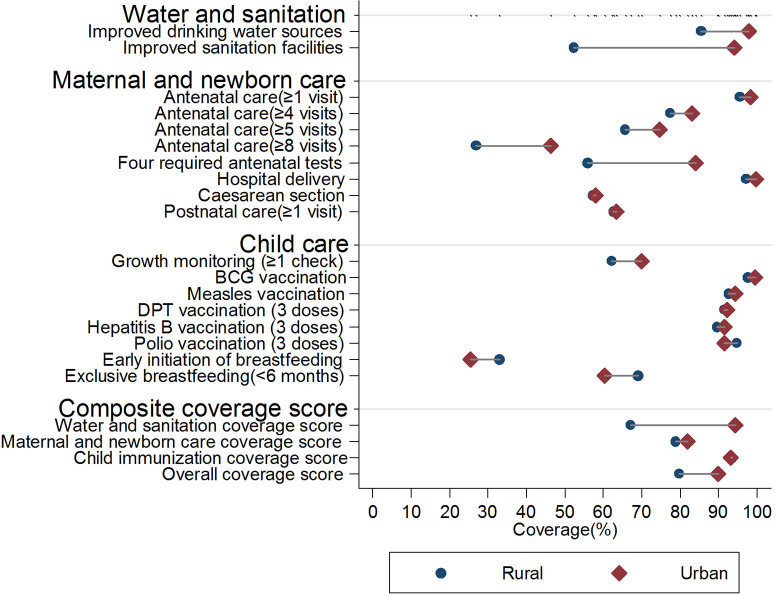
Equity gaps between rural and urban settings in essential MNCH interventions, Jilin province, China (2008–2018). For details of the regression results for each specific intervention, please see Appendix 4.
In the water and sanitation domain, the interventions were adjusted for setting, household economic status, time to the nearest health facility, household head educational achievement, health insurance coverage, and ethnicity.In the maternal and newborn domain, the interventions were adjusted for setting, household economic status, time to the nearest health facility, mother's educational achievement, health insurance coverage, age when giving birth, parity, and ethnicity.In the child care domain, the interventions were adjusted for setting, household economic status, household head educational achievement, time to the nearest health facility, children's health insurance, age in months, sex, and ethnicity.Hospital delivery and BCG vaccination were not adjusted because the proportions were near 100%.

**Figure 5:**
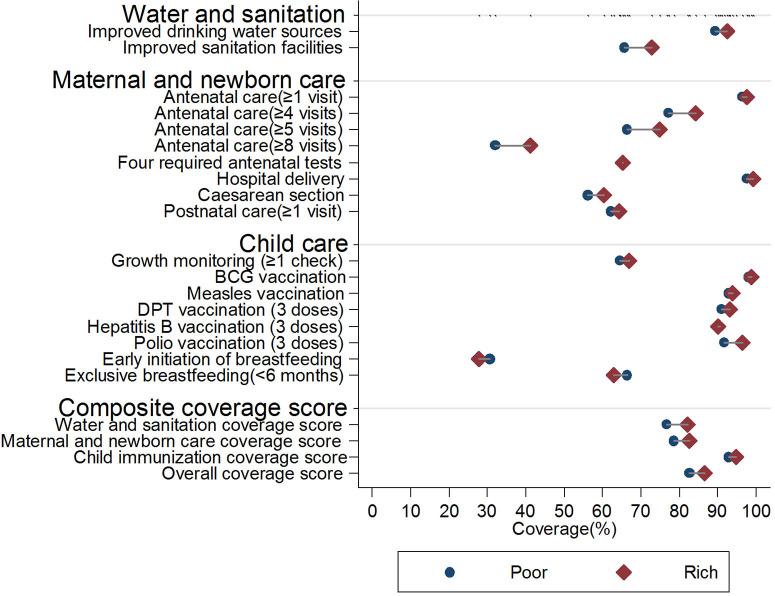
Equity gaps between poor and rich essential MNCH interventions, Jilin province, China (2008–2018). For details of the regression results for each specific intervention, please see Appendix 4.
In the water and sanitation domain, the interventions were adjusted for setting, household economic status, time to the nearest health facility, household head educational achievement, health insurance coverage, and ethnicity.In the maternal and newborn domain, the interventions were adjusted for setting, household economic status, time to the nearest health facility, mother's educational achievement, health insurance coverage, age when giving birth, parity, and ethnicity.In the child care domain, the interventions were adjusted for settings, household economic status, household head educational achievement, time to the nearest health facility, children's health insurance, age in months, sex, and ethnicity.Hospital delivery and BCG vaccination were not adjusted because the proportions were near 100%.

**Figure 6:**
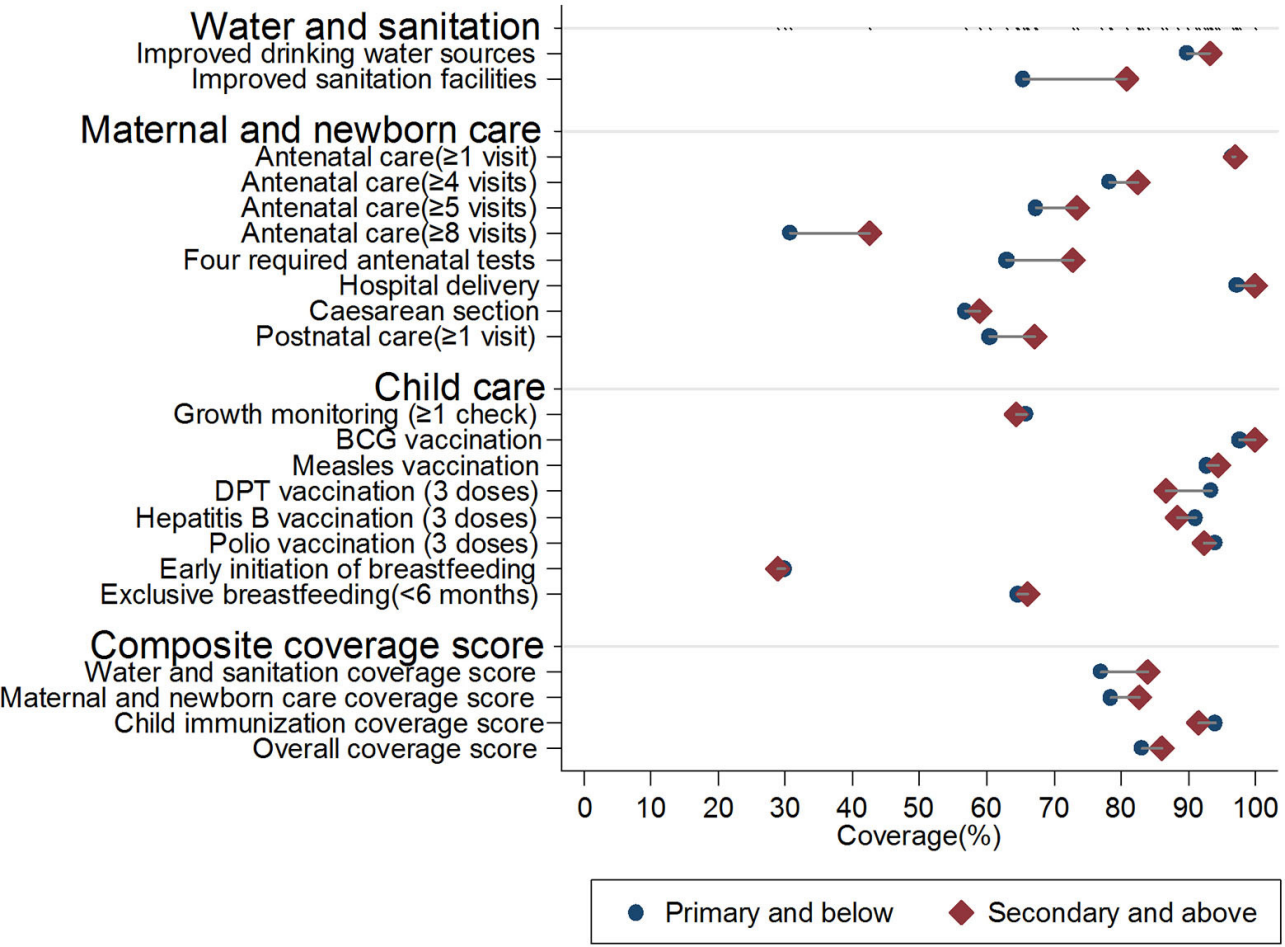
Equity gaps between low and high educational achievements in essential MNCH interventions, Jilin province, China (2008–2018). For details of the regression results for each specific intervention, please see Appendix 4.
In the water and sanitation domain, the interventions were adjusted for setting, household economic status, time to the nearest health facility, household head educational achievement, health insurance coverage, and ethnicity.In the maternal and newborn domain, the interventions were adjusted for setting, household economic status, time to the nearest health facility, mother's educational achievement, health insurance coverage, age when giving birth, parity, and ethnicity.In the child care domain, the interventions were adjusted for setting, household economic status, household head educational achievement, time to the nearest health facility, children's health insurance, age in months, sex, and ethnicity.Hospital delivery and BCG vaccination were not adjusted because the proportions were near 100%.

Figures 4–6 illustrate the equity gaps for the 18 essential MNCH interventions and the composite coverage scores between urban and rural settings, poor and rich households, and women with above and below primary education (all these values were predicted by logistic regression models adjusting for other covariates. For details of the regression analyses, please see Appendix 4). The results reveal different types of inequity in different domains. In the water and sanitation domain, substantial rural–urban equity gaps exist for coverage of improved drinking water sources (85.4 vs. 97.9%, *p* < 0.01) and improved sanitation facilities (52.5 vs. 94.2%, *p* < 0.01), whereas the adjusted equity gaps were relatively small across rich vs. poor and household heads with high vs. low educational achievement. In the maternal and newborn care domain, most interventions had similarly small equity gaps except for four government-funded antenatal care services (55.8 vs. 84.1% for rural vs. urban, *p* < 0.01) and at least eight antenatal care sessions (26.8 vs. 46.3% for rural vs. urban, *p* < 0.05). In the child care domain, most of the differences were either small in scale or statistically insignificant.

Table 2 shows the factors associated with the composite coverage scores and the trends. For the water and sanitation coverage score, the adjusted urban–rural difference was 27.3% (95% CI 22.3–32.3%), whereas the effects of household-level economic status and educational achievement were substantially attenuated by other factors (adjusted differences 5.4%, 95% CI 3.3–7.5% comparing rich and poor and 6.6%, 95% CI 4.2–9.0% comparing above and below primary education). For maternal and newborn care coverage scores, the adjusted urban–rural, poor–rich, and high–low educational differences were relatively small. In addition, mothers who could access a health facility in 15 min had higher composite coverage scores than those who could access a health facility in more than 15 min (adjusted difference 9.4%, 95% CI 2.7–16.1%).

**Table 2:** Factors associated with the composite coverage scores for essential MNCH interventions in Jilin province, China (2008–2018).

**Characteristics**	**Water and sanitation coverage score^a^**			**Maternal and newborn care coverage score^b^**			**Child immunization coverage score^c^**		
	**Score**	**Unadjusted difference**	**Adjusted difference**	**Score**	**Unadjusted difference**	**Adjusted difference**	**Score**	**Unadjusted difference**	**Adjusted difference**
	**(%)**	**[95% CI]**	**[95% CI]**	**(%)**	**[95% CI]**	**[95% CI]**	**(%)**	**[95% CI]**	**[95% CI]**
**Year of survey**									
2008	75.4	Reference	Reference	74.7	Reference	Reference	94.4	Reference	Reference
2013	84.3	8.9^*^	9.5^**^	76.4	1.6	1.2	92.1	−2.3	−3.9^*^
		[1.5, 16.3]	[3.6, 15.3]		[−5.7, 9.0]	[−5.7, 8.2]		[−5.7, 1.2]	[−7.3, −0.5]
2018	77.4	1.9	0.2	90.1	15.4^**^	12.1^**^			
		[−6.4, 10.3]	[−6.2, 6.5]		[9.3, 21.5]	[6.3, 17.9]			
**Setting**									
Rural	65.0	Reference	Reference	76.9	Reference	Reference	93.9	Reference	Reference
Urban	96.9	31.8^**^	27.3^**^	84.2	7.2^**^	1.1	92.1	−1.8	0.4
		[27.0, 36.6]	[22.3, 32.3]		[2.2, 12.3]	[−4.5, 6.8]		[−5.6, 2.0]	[−3.7, 4.5]
**Economic status**									
Poor	71.0	Reference	Reference	77.1	Reference	Reference	93.0	Reference	Reference
Rich	88.2	17.2^**^	5.4^**^	84.7	7.6^**^	3.7^*^	93.8	0.8	1.5
		[14.2, 20.2]	[3.3, 7.5]		[4.3, 11.0]	[0.7, 6.8]		[−2.2, 3.7]	[−1.1, 4.0]
**Educational achievement**									
Primary and below	72.0	Reference	Reference	77.0	Reference	Reference	93.9	Reference	Reference
Secondary and above	93.9	21.9^**^	6.6^**^	85.0	8.1^**^	2.5	91.3	−2.6	−2.2
		[18.8, 25.0]	[4.2, 9.0]		[4.0, 12.1]	[−1.3, 6.4]		[−6.2, 1.1]	[−5.6, 1.2]
**Time to the nearest health facility**									
> 15 min	72.2	Reference	Reference	69.3	Reference	Reference	88.8	Reference	Reference
≤15 min	80.6	8.4^**^	4.2	81.5	12.2^**^	9.4^**^	94.3	5.4	5.0
		[2.7, 14.2]	[−0.2, 8.5]		[5.1, 19.2]	[2.7, 16.1]		[−0.4, 11.3]	[−0.8, 10.7]
**Health insurance coverage**									
No	84.6	Reference	Reference	75.1	Reference	Reference	90.7	Reference	Reference
Yes	78.7	−5.8^**^	0.6	81.0	5.9^*^	4.7	94.6	3.9^*^	4.9^**^
		[−9.6, −2.1]	[−2.4, 3.5]		[0.9, 11.0]	[−0.7, 10.0]		[0.6, 7.1]	[1.7, 8.1]
**N of observations**			16,718			1,530			921
***F*****-value**			34.1			7.53			2.0
***P*****-value**			<0.0001			<0.0001			0.0308
***R***^**2**^			0.3211			0.1045			0.0412

a *Adjusting for survey year, setting, economic status, time to the nearest health facility, household head's educational achievement, health insurance coverage, and ethnicity*.

b *Adjusting for survey year, setting, economic status, time to the nearest health facility, mother's educational achievement, health insurance coverage, age when giving birth, parity, and ethnicity*.

c *Adjusting for survey year, setting, economic status, household head's educational achievement, time to the nearest health facility, children's health insurance coverage, age in months, sex, and ethnicity*.

* p < 0.05;

** *p < 0.01*.

The changing trends were particularly evident among those living in rural settings. When adjusting for other covariates, the water and sanitation coverage score increased by 14.1 percentage points (95% CI 4.7–23.6%) from 2008 to 2013 but returned to the 2008 level in 2018, which made the rural–urban gap first shrink and then expand. For maternal and newborn care coverage, a significant rising trend occurred (adjusted differences 12.1%, 95% CI 6.3–17.9% comparing 2008 and 2018). For figure on the trends, please see Appendix 5.

## Discussion

Using provincially representative survey data, we reported the coverage and equity of 18 interventions along the MNCH care continuum in Jilin province, China, during 2008 and 2018. Given its size and socioeconomic development level, Jilin province accommodates the population of a middle-level country globally and can be considered a typical region in China. We found high coverage of hospital-based interventions in this region. Some interventions, such as cesarean section, presented alarmingly high rates—among the highest rates worldwide—indicating over-coverage (33). However, substantial unmet needs exist in many community-based interventions that are delivered in primary care settings or rely on wider systems, such as antenatal and postnatal care, child growth monitoring, child feeding, and water and sanitation. Such inequity mainly presented as urban–rural disparities rather than household- or individual-level inequalities across people's income and educational status, especially in the water and sanitation domain and quality coverage of maternal and newborn care interventions.

Compared to other LMICs, the main difference is that, in other resource-insufficient countries, improvements have mainly involved scaling up community-based MNCH interventions although substantial gaps persist in intrapartum care and other hospital-based interventions (1, 7, 34–37). The findings demonstrate China's health system development process and hospital-based strategy to improve maternal and newborn care in recent decades.

In the 1960s−1980s, China built a community-oriented and integrated health system that focused on effectively linked three-tier systems, barefoot doctors, and community-based financing mechanisms. The system provides information for many LIMCs due to its ability to provide essential health services with high cost-effectiveness given resource constraints (19). However, China moved away from this model after the economic reform in the 1980s. Decentralization provided more autonomy as well as profit-making pressure on hospitals. Users were given freedom to choose public hospitals as their first contact, and resources were redistributed among different health service providers. Great achievements were made in constructing many modern advanced hospitals. However, there are two concerns about such a paradigm shift. First, boosted by an increasingly common belief in science and technology and distorted by incentives to maximize revenue, Chinese hospitals were considered to be part of a “medical arms race” that focused on developing high-technology and costly examinations and procedures, which might usher in the overuse of curative care and uncontrolled rising medical expenditures (18, 20, 38). Second, the promotion of market-oriented financing mechanisms changed providers' relationships from collaborative to competitive (39, 40). In the competition with advanced and profitable hospital care, community-based care was gradually marginalized (41). Consequently, the primary care system that used to provide collaborative and continuing care was dismantled in China in the 1990s (18, 19, 27).

We found that hospital-based interventions, such as hospital delivery and antenatal B-ultrasound examination, were almost universal in Jilin province, China. On the one hand, the cesarean section rate was as high as 51.8% in 2008 and increased to 64.9% in 2013, similar to the rates reported from the government's annual report data (42). In our study, most mothers were primiparas and gave birth at <30 years of age. Considering such a high level of prevalence and low risk, the high cesarean section rate could certainly be seen as an indication of over-medicalization. By contrast, most community-based interventions that are delivered in primary care settings, such as antenatal and postpartum care as well as child growth monitoring, presented low coverage and revealed unmet needs. For example, the proportions of mothers who received at least one session of postnatal care were lower than 65%, and coverage of at least four sessions of antenatal care was no higher than 80%. Unlike hospital delivery, these services warrant lower requirements for equipment and personnel skills and could be effectively delivered by township and village health providers.

Especially for water and sanitation and the quality coverage of maternal and newborn care interventions, urban–rural inequalities were more profound than individual-level disparities across ethnicity, education, income, and health insurance status. These patterns are different from other *Countdown* countries, where social status, mother's educational achievement, and families' ability to pay are considered to be main barriers in accessing quality MNCH care (43–47). Jilin province has moderate economic development and ranked 11th out of 31 provinces in China in terms of rural household per capita income (48). In our study, most of the mothers achieved primary education and above, which means they have the ability to acquire basic health information and make health decisions. These findings suggest that, once basic wealth and education are guaranteed, individual-level social determinants may be less important barriers that undermine access to essential MNCH services, and regional-level system endowment or deficiency might have a more considerable impact.

Encouragingly, coverage of maternal and newborn care interventions was improved and the inequity shrank substantially during 2008–2018, such as the four types of government-funded antenatal care services (from 50.4% in 2008 to 82.2% in 2013) and at least five antenatal care sessions (from 61.6% in 2008 to 85.4% in 2018). These interventions were included in China's *National Essential Public Health Services* program initiated in 2009. In the program, the government offers free access to antenatal and postnatal care, growth monitoring, and maternal health education as well as free vaccinations for BCG, measles, DPT, hepatitis B, and polio (24–26). Special funds are allocated to purchase these services from community health care providers on a per capita basis. The government also made additional efforts to improve rural infrastructures. In contrast, water and sanitation involved more multi-sector coordination interventions and had no specific subsidy, which made their improvement more difficult (49, 50). However, even in the program, improvements were not observed in all interventions, and sluggish coverage was observed for interventions such as at least one session of growth monitoring and child feeding. Taken together, these results suggest that China's recent efforts to revitalize its primary health care system are partly effective in reducing health inequity. In fact, although the reform made efforts to strengthen the primary care system, hospitals still perform nearly 50% of outpatient services (48).

The data and analysis in this study warrant scrutiny. First, the Jilin province NHSS is provincially representative. Given the size, socioeconomic situation, and health system development in Jilin province, the findings can somewhat depict the situation in China. However, China is very large, and the situation in the western part is quite different from other regions (12). Second, we built on the *Countdown* approach but made some changes due to the data and China's specific contexts. For example, we analyzed hospital delivery instead of skilled birth attendants and postnatal care within 42 days instead of 2 days. Unlike many international health surveys, such as the Demographic and Health Surveys (DHSs), which only surveyed children aged 12–23 months for vaccination coverage, children 12–59 months were used to define vaccination coverage in this analysis to maximize the sample size. We performed a sensitivity analysis and found no changes for the findings (Appendix 6). Furthermore, we added some interventions that were not included in the *Countdown*, such as at least eight antenatal care sessions, four types of government-funded antenatal services, BCG vaccination, hepatitis B vaccination (three doses), polio vaccination (three doses), and growth monitoring (≥1 check). These indicators are included in either the WHO 2016 recommendations on quality antenatal care services or China's *National Essential Public Health Services* program. Third, China's NHSS provided very limited information in performing the same principal component analysis as the DHS (51). Thus, we used household annual income per capita instead of the wealth index as used in the *Countdown* for equity analysis. Fourth, recall bias may occur in the household surveys, but we believe the coverage and equity of interventions were still comparable among different years since the same survey method was used.

Other LIMCs could draw lessons from China. The obstetrician-led MNCH care strategy may come at the cost of over-medicalization and health inequity. Taking Jilin province as an example, we also find that recent efforts in China to revitalize primary health care exhibit the potential for change. An integrated system that links families, communities, and all levels of health care organizations seems to be the most effective and efficient model to offer continuing MNCH care.

## Data Availability Statement

The data analyzed in this study was obtained from the Jilin Health Information and Statistics Center. No individual private information could be released to the public domain. Requests to access these datasets should be directed to Yaoguang Zhang, zhangyg@nhfpc.gov.cn.

## Ethics Statement

Ethical review and approval was not required for the study on human participants in accordance with the local legislation and institutional requirements. Written informed consent for participation was not required for this study in accordance with the national legislation and the institutional requirements.

## Author Contributions

XF conceived the study. YW did the analysis and made the first draft under XF's supervision. RL helped to refine the report and XF finalized the report. All authors contributed to critical interpretation of the results and development of the report, and approved the final version.

## Conflict of Interest

The authors declare that the research was conducted in the absence of any commercial or financial relationships that could be construed as a potential conflict of interest.
