# Supplementary material for: Equity in Essential Maternal, Newborn, and Child Health Interventions in Northeastern China, 2008 to 2018
Source: Front Public Health. 2020 Jul 2;8:212. doi: 10.3389/fpubh.2020.00212 (PMC7343890; doi:10.3389/fpubh.2020.00212)
Supplement: Supplementary file 1 [file Data_Sheet_1.docx]

**Appendix 1. Location and socio-demographic profiles of Jilin province, China**

- 1. **Location of Jilin province in China (separated by province boarder, Jilin province in dark green)**


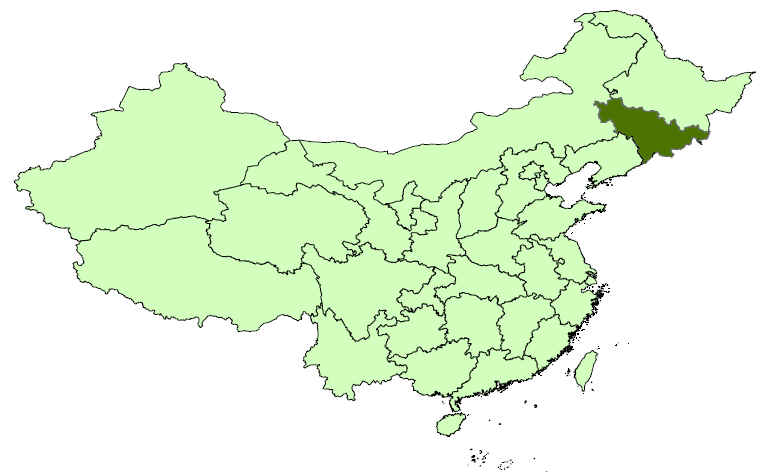


**1.2 Jilin province and China’s national profiles in socioeconomic and health system development (data for the year 2013)**

|  | **China’s national profiles** | **Jilin province’s profiles** |
| --- | --- | --- |
| **Land area (1,000 km^2^)** | 960.0 | 18.7 |
| **Total population (million)** | 1360.7 | 27.5 |
| **U5MR in 1990 (per thousand live births)** | 59.1 | 32.3 |
| **U5MR in 2013 (per thousand live births)** | 13.0 | 8.4 |
| **Percentage of U5MR reduction from 1990 to 2013 (%)** | 78.0 | 74.0 |
| **Per capita GDP ($)** | 6,904 | 7,774 |
| **Proportion of urban population (%)** | 53.7 | 54.2 |
| **Proportion of illiterate population (%)** | 4.6 | 2.3 |
| **Per capita total health expenditure ($)** | 383 | 458 |
| **Density of hospital beds (per 10,000 population)** | 4.6 | 4.8 |
| **Density of total health professionals (per 1,000 population)** | 5.3 | 5.5 |

Notes: Under 5 mortality rate (U5MR) data were from estimates of Wang, etc.^1^ (<https://vizhub.healthdata.org/subnational/china>), and other data were from China Statistical Yearbook 2014^2^.

**Appendix 2. Sampling information for** **NHSS Jilin province**

**2.1 Sampling households and population**

|  | **Households** | |  | **Population** | |
| --- | --- | --- | --- | --- | --- |
|  | **total** | **sampled** |  | **total** | **sampled** |
| 2008 | 8,849,300 | 4,778 |  | 26,960,400 | 13,922 |
| 2013 | 9,781,300 | 6,000 |  | 27,015,000 | 15,573 |
| 2018 | 10,202,100 | 6,000 |  | 26,157,900 | 16,759 |

**2.2 Counties sampled in the 2008 survey（separated by county borders, sampled counties in dark green）**


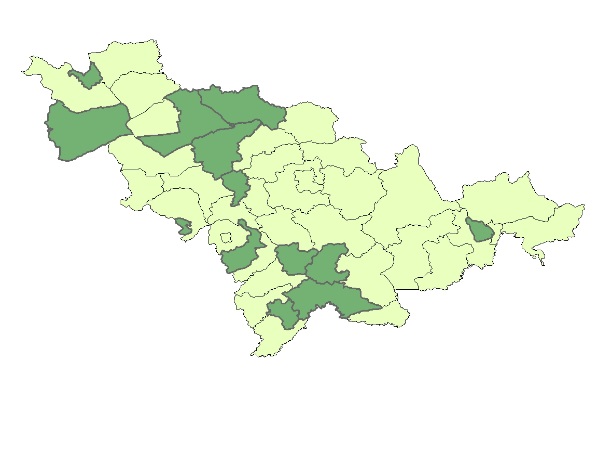


**2.3 Counties sampled in the 2013 and 2018 survey（separated by county borders, sampled counties in dark green）**


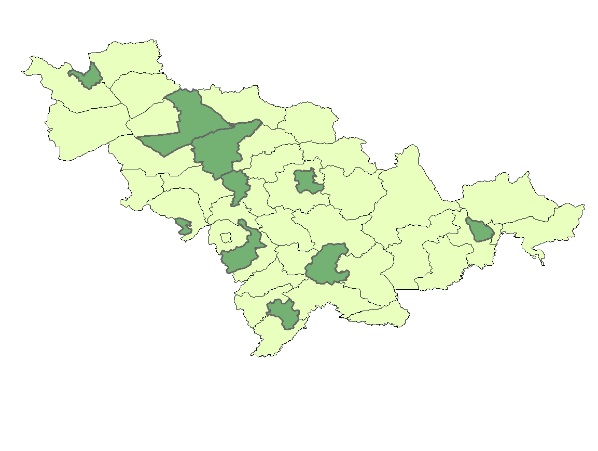


**Appendix 3. Correlations between different composite coverage scores and child health outcomes**

Notes：the composite coverage scores and the child health outcomes were all at the county level. Under five mortality rate data were from estimates of Wang, etc.^1^ (<https://vizhub.healthdata.org/subnational/china>) and stunting rate were calculated by the survey data using the WHO definition and analysing macro for Stata. (<https://www.who.int/childgrowth/software/en/>)

**Appendix 4. Factors associated with the coverage of specific essential MNCH interventions**

**4.1 Factors associated with the coverage of water and sanitation interventions in Jilin province, China (2008-2018)**

| **Characteristics** | **Improved drinking water sources** | |  | **Improved sanitation facilities** | |
| --- | --- | --- | --- | --- | --- |
|  | **Adjusted OR** | **95% CI** |  | **Adjusted OR** | **95% CI** |
|  |  |  |  |  |  |
| **Settings** |  |  |  |  |  |
| Rural | 1 |  |  | 1 |  |
| Urban | 7.97* | [3.67,17.31] |  | 16.78* | [8.67,32.47] |
| **Household head’s ethnicity** |  |  |  |  |  |
| Minority | 1 |  |  | 1 |  |
| Majority | 1.68 | [0.92,3.06] |  | 0.90 | [0.59,1.37] |
| **Economic status** |  |  |  |  |  |
| Poor | 1 |  |  | 1 |  |
| Rich | 1.49* | [1.14,1.94] |  | 1.61* | [1.30,1.98] |
| **Household head’s educational achievement** |  |  |  |  |  |
| Primary and below | 1 |  |  | 1 |  |
| Secondary and above | 1.56* | [1.12,2.17] |  | 2.79* | [2.14,3.65] |
| **Time to the nearest health facility** |  |  |  |  |  |
| >15 min | 1 |  |  | 1 |  |
| ≤15 min | 1.13 | [0.63,2.02] |  | 1.30 | [0.92,1.85] |
| **Household head’s health insurance coverage** |  |  |  |  |  |
| No | 1 |  |  | 1 |  |
| Yes | 1.85* | [1.24,2.76] |  | 1.18 | [0.86,1.61] |

**4.2-1 Factors associated with the coverage of maternal and newborn care interventions, Jilin province, China (2008-2018)**

| **Characteristics** | **Antenatal care** | |  | **Antenatal care** | |  | **Antenatal care** | |  | **Antenatal care** | |
| --- | --- | --- | --- | --- | --- | --- | --- | --- | --- | --- | --- |
|  | **(≥1 visit)** | |  | **(≥4 visits)** | |  | **(≥5 visits)** | |  | **(≥8 visits)** | |
|  | **Adjusted OR** | **95% CI** |  | **Adjusted OR** | **95% CI** |  | **Adjusted OR** | **95% CI** |  | **Adjusted OR** | **95% CI** |
| **Age giving birth** |  |  |  |  |  |  |  |  |  |  |  |
| <20 years old | 1 |  |  | 1 |  |  | 1 |  |  | 1 |  |
| 20-23 years old | 0.92 | [0.17,5.12] |  | 1.35 | [0.64,2.85] |  | 1.22 | [0.58,2.56] |  | 1.25 | [0.57,2.72] |
| 24-34 years old | 1.05 | [0.20,5.54] |  | 1.78 | [0.81,3.89] |  | 1.45 | [0.69,3.05] |  | 1.70 | [0.76,3.83] |
| >=35 years old | 0.68 | [0.12,3.87] |  | 1.39 | [0.60,3.22] |  | 1.24 | [0.56,2.77] |  | 1.54 | [0.65,3.67] |
| **Parity** |  |  |  |  |  |  |  |  |  |  |  |
| >1 | 1 |  |  | 1 |  |  | 1 |  |  | 1 |  |
| 1 | 0.75 | [0.38,1.51] |  | 1.03 | [0.74,1.44] |  | 1.13 | [0.85,1.52] |  | 1.12 | [0.85,1.48] |
| **Ncd** |  |  |  |  |  |  | |  |  |  |  |
| No | 1 |  |  | 1 |  |  | 1 |  |  | 1 |  |
| Yes | 2.51 | [0.33,19.28] |  | 1.87 | [0.96,3.64] |  | 1.25 | [0.78,2.00] |  | 1.59 | [0.94,2.69] |
| **Settings** |  |  |  |  |  |  |  |  |  |  |  |
| Rural | 1 |  |  | 1 |  |  | 1 |  |  | 1 |  |
| Urban | 2.97 | [0.92,9.62] |  | 1.45 | [0.85,2.48] |  | 1.57 | [0.96,2.57] |  | 2.50* | [1.58,3.96] |
| **Ethnicity** |  |  |  |  |  |  | |  |  |  |  |
| Minority | 1 |  |  | 1 |  |  | 1 |  |  | 1 |  |
| Majority | 1.00 | [0.36,2.73] |  | 0.36* | [0.18,0.73] |  | 0.44* | [0.27,0.74] |  | 0.76 | [0.46,1.24] |
| **Economic status** |  |  |  |  |  |  |  |  |  |  |  |
| Poor | 1 |  |  | 1 |  |  | 1 |  |  | 1 |  |
| Rich | 1.45 | [0.72,2.94] |  | 1.63* | [1.15,2.31] |  | 1.54* | [1.13,2.09] |  | 1.58* | [1.24,2.03] |
| **Educational achievement** |  |  |  |  |  |  |  |  |  |  |  |
| Primary and below | 1 |  |  | 1 |  |  | 1 |  |  | 1 |  |
| Secondary and above | 1.13 | [0.59,2.16] |  | 1.32 | [0.88,1.97] |  | 1.37 | [0.97,1.93] |  | 1.77* | [1.26,2.49] |
| **Time to the nearest health facility** |  |  |  |  |  |  |  |  |  |  |  |
| >15 min | 1 |  |  | 1 |  |  | 1 |  |  | 1 |  |
| ≤15 min | 0.95 | [0.34,2.68] |  | 2.38* | [1.46,3.88] |  | 2.05* | [1.27,3.30] |  | 1.97* | [1.24,3.14] |
| **Health insurance coverage** |  |  |  |  |  |  |  |  |  |  |  |
| No | 1 |  |  | 1 |  |  | 1 |  |  | 1 |  |
| Yes | 2.72 | [0.78,9.46] |  | 1.83* | [1.15,2.93] |  | 1.85* | [1.21,2.82] |  | 1.70* | [1.02,2.82] |

**4.2-2 Factors associated with the coverage of maternal and newborn care interventions, Jilin province, China (2008-2018)**

| **Characteristics** | **Four government-funded antenatal services** | |  | **Caesarean section** | |  | **Postnatal care** | |
| --- | --- | --- | --- | --- | --- | --- | --- | --- |
|  |  |  |  |  | |  | **(≥1 visit)** | |
|  | **Adjusted OR** | **95% CI** |  | **Adjusted OR** | **95% CI** |  | **Adjusted OR** | **95% CI** |
| **Age giving birth** |  |  |  |  |  |  |  |  |
| <20 years old | 1 |  |  | 1 |  |  | 1 |  |
| 20-23 years old | 0.70 | [0.32,1.53] |  | 1.06 | [0.54,2.09] |  | 1.20 | [0.56,2.55] |
| 24-34 years old | 0.78 | [0.35,1.71] |  | 1.32 | [0.66,2.61] |  | 1.32 | [0.63,2.78] |
| >=35 years old | 1.00 | [0.42,2.37] |  | 1.68 | [0.80,3.55] |  | 1.20 | [0.53,2.70] |
| **Parity** |  |  |  |  |  |  |  |  |
| >1 | 1 |  |  | 1 |  |  | 1 |  |
| 1 | 1.48* | [1.00,2.19] |  | 1.38* | [1.08,1.77] |  | 0.81 | [0.62,1.06] |
| **Ncd** |  |  |  |  |  |  |  |  |
| No | 1 |  |  | 1 |  |  | 1 |  |
| Yes | 0.76 | [0.36,1.58] |  | 1.34 | [0.81,2.19] |  | 1.00 | [0.62,1.62] |
| **Settings** |  |  |  |  |  |  |  |  |
| Rural | 1 |  |  | 1 |  |  | 1 |  |
| Urban | 4.39* | [2.54,7.57] |  | 1.03 | [0.74,1.42] |  | 1.04 | [0.61,1.75] |
| **Ethnicity** |  |  |  |  |  |  |  |  |
| Minority | 1 |  |  | 1 |  |  | 1 |  |
| Majority | 0.46* | [0.26,0.80] |  | 0.69 | [0.47,1.02] |  | 0.95 | [0.62,1.45] |
| **Economic status** |  |  |  |  |  |  |  |  |
| Poor | 1 |  |  | 1 |  |  | 1 |  |
| Rich | 0.99 | [0.70,1.42] |  | 1.19 | [0.94,1.51] |  | 1.11 | [0.84,1.46] |
| **Educational achievement** |  |  |  |  |  |  |  |  |
| Primary and below | 1 |  |  | 1 |  |  | 1 |  |
| Secondary and above | 1.67* | [1.06,2.61] |  | 1.09 | [0.82,1.45] |  | 1.36 | [0.94,1.97] |
| **Time to the nearest health facility** |  |  |  |  |  |  |  |  |
| >15 min | 1 |  |  | 1 |  |  | 1 |  |
| ≤15 min | 1.22 | [0.77,1.94] |  | 1.12 | [0.80,1.56] |  | 1.57* | [1.02,2.40] |
| **Health insurance coverage** |  |  |  |  |  |  |  |  |
| No | 1 |  |  | 1 |  |  | 1 |  |
| Yes | 1.65* | [1.05,2.59] |  | 0.99 | [0.71,1.39] |  | 1.81* | [1.31,2.50] |

**4.3-1 Factors associated with the coverage of children’s interventions, Jilin province, China (2008-2018)**

| **Characteristics** | **Growth monitoring**  **(≥1 check)** | |  | **Measles vaccination** | |  | **DPT vaccination**  **(3 doses)** | |  | **Hepatitis B vaccination**  **(3 doses)** | |
| --- | --- | --- | --- | --- | --- | --- | --- | --- | --- | --- | --- |
|  |  |  |  |  |  |  |  |  |  |  |  |
|  | **Adjusted OR** | **95% CI** |  | **Adjusted OR** | **95% CI** |  | **Adjusted OR** | **95% CI** |  | **Adjusted OR** | **95% CI** |
| **Age in month** |  |  |  |  |  |  |  |  |  |  |  |
| 0-11m | 1 |  |  | - | - |  | - | - |  | - | - |
| 12-23m | 0.83 | [0.60,1.16] |  | 1 |  |  | 1 |  |  | 1 |  |
| 24-35m | 0.56* | [0.39,0.83] |  | 0.57 | [0.28,1.18] |  | 1.30 | [0.66,2.57] |  | 0.95 | [0.56,1.59] |
| 36-47m | 0.58* | [0.40,0.84] |  | 0.80 | [0.40,1.63] |  | 1.05 | [0.52,2.11] |  | 0.91 | [0.48,1.69] |
| 48-59m | 0.40* | [0.28,0.56] |  | 0.58 | [0.28,1.21] |  | 1.06 | [0.55,2.06] |  | 1.09 | [0.58,2.05] |
| **Sex** |  |  |  |  |  |  |  |  |  |  |  |
| Female | 1 |  |  | 1 |  |  | 1 |  |  | 1 |  |
| Male | 0.98 | [0.80,1.21] |  | 1.27 | [0.69,2.33] |  | 0.85 | [0.52,1.40] |  | 0.99 | [0.60,1.62] |
| **Ethnicity** |  |  |  |  |  |  |  |  |  |  |  |
| Minority | 1 |  |  | 1 |  |  | 1 |  |  | 1 |  |
| Majority | 0.86 | [0.50,1.45] |  | 2.05* | [1.04,4.05] |  | 0.70 | [0.21,2.29] |  | 0.91 | [0.40,2.05] |
| **Settings** |  |  |  |  |  |  |  |  |  |  |  |
| Rural | 1 |  |  | 1 |  |  | 1 |  |  | 1 |  |
| Urban | 1.43 | [0.91,2.26] |  | 1.32 | [0.57,3.06] |  | 1.08 | [0.49,2.39] |  | 1.26 | [0.50,3.13] |
| **Economic status** |  |  |  |  |  |  |  |  |  |  |  |
| Poor | 1 |  |  | 1 |  |  | 1 |  |  | 1 |  |
| Rich | 1.12 | [0.81,1.53] |  | 1.17 | [0.66,2.09] |  | 1.38 | [0.75,2.52] |  | 0.95 | [0.53,1.71] |
| **Household head's educational achievement** | | | | | | | | | | | |
| Primary and below | 1 |  |  | 1 |  |  | 1 |  |  | 1 |  |
| Secondary and above | 0.93 | [0.70,1.24] |  | 1.36 | [0.64,2.90] |  | 0.45* | [0.24,0.85] |  | 0.73 | [0.40,1.32] |
| **Time to the nearest health facility** | | | | | | | | | | | |
| > 15 min | 1 |  |  | 1 |  |  | 1 |  |  | 1 |  |
| ≤15 min | 1.18 | [0.78,1.78] |  | 1.37 | [0.70,2.69] |  | 2.30* | [1.09,4.86] |  | 2.19* | [1.06,4.52] |
| **Health insurance coverage** | | | | | | | | | | | |
| No | 1 |  |  | 1 |  |  | 1 |  |  | 1 |  |
| Yes | 0.95 | [0.69,1.31] |  | 1.12 | [0.55,2.29] |  | 1.60 | [0.87,2.94] |  | 1.63 | [0.81,3.27] |

**4.3-2 Factors associated with the coverage of children’s interventions, Jilin province, China (2008-2018)**

| **Characteristics** | **Polio vaccination** | |  | **Early initiation of breastfeeding** | |  | **Exclusive breastfeeding within 6 months** | |
| --- | --- | --- | --- | --- | --- | --- | --- | --- |
|  | **(3 doses)** | |  |  |  |  |  |  |
|  | **Adjusted OR** | **95% CI** |  | **Adjusted OR** | **95% CI** |  | **Adjusted OR** | **95% CI** |
| **Age in month** |  |  |  |  |  |  |  |  |
| 0-11m | - | - |  | 1 |  |  | 1 |  |
| 12-23m | 1 |  |  | 1.00 | [0.65,1.51] |  | 0.95 | [0.62,1.47] |
| 24-35m | 0.78 | [0.38,1.59] |  | 1.26 | [0.80,1.98] |  | 1.30 | [0.79,2.16] |
| 36-47m | 1.45 | [0.61,3.46] |  | 1.49 | [0.93,2.37] |  | 0.96 | [0.62,1.49] |
| 48-59m | 1.68 | [0.78,3.64] |  | 0.93 | [0.61,1.42] |  | 1.13 | [0.72,1.78] |
| **Sex** |  |  |  |  |  |  |  |  |
| Female | 1 |  |  | 1 |  |  | 1 |  |
| Male | 0.87 | [0.51,1.47] |  | 0.85 | [0.65,1.11] |  | 0.88 | [0.67,1.16] |
| **Ethnicity** |  |  |  |  |  |  |  |  |
| Minority | 1 |  |  | 1 |  |  | 1 |  |
| Majority | 0.71 | [0.22,2.28] |  | 1.63 | [0.94,2.80] |  | 1.17 | [0.78,1.76] |
| **Settings** |  |  |  |  |  |  |  |  |
| Rural | 1 |  |  | 1 |  |  | 1 |  |
| Urban | 0.57 | [0.22,1.49] |  | 0.69 | [0.40,1.22] |  | 0.68* | [0.51,0.91] |
| **Economic status** |  |  |  |  |  |  |  |  |
| Poor | 1 |  |  | 1 |  |  | 1 |  |
| Rich | 2.57* | [1.16,5.68] |  | 0.87 | [0.57,1.33] |  | 0.86 | [0.64,1.15] |
| **Household head's educational achievement** | | | | | | | | |
| Primary and below | 1 |  |  | 1 |  |  | 1 |  |
| Secondary and above | 0.75 | [0.33,1.70] |  | 0.95 | [0.63,1.42] |  | 1.07 | [0.73,1.56] |
| **Time to the nearest health facility** | | | | | | | | |
| > 15 min | 1 |  |  | 1 |  |  | 1 |  |
| ≤15 min | 2.42* | [1.01,5.80] |  | 1.20 | [0.58,2.47] |  | 0.98 | [0.66,1.47] |
| **Health insurance coverage** | | | | | | | | |
| No | 1 |  |  | 1 |  |  | 1 |  |
| Yes | 1.67 | [0.82,3.37] |  | 1.18 | [0.84,1.67] |  | 1.24 | [0.95,1.61] |

**Appendix 5. Trends in composite coverage scores for essential MNCH interventions in Jilin province, China (2008-2018), by setting, economic status and educational achievement**

**Notes:**

a-The water and sanitation coverage score was adjusting for survey year, settings, household’s economic status, time to the nearest health facility, household head’s educational achievement, health insurance coverage, and ethnicity.

b-The maternal and newborn care coverage score was adjusting for survey year, settings, household’s economic status, time to the nearest health facility, mother’s educational achievement, health insurance coverage, age when gave birth, parity, and ethnicity.

c-The child immunization coverage score was adjusting for survey year, settings, household’s economic status, household head’s educational achievement, time to the nearest health facility, children’s health insurance coverage, age in month, sex, and ethnicity.

**Appendix 6. Sensitivity analysis for vaccination coverage, comparing the sample of 12-59 months old children with those 12-23 months old, Jilin province, China (2008-2013)**

|  | **2008** | | | |  | **2013** | | | |
| --- | --- | --- | --- | --- | --- | --- | --- | --- | --- |
|  | **12-59 months** | **12-23 months** | ***χ^2^*** | **P-value** |  | **12-59 months** | **12-23 months** | ***χ^2^*** | **P-value** |
| **BCG vaccination (%)** | 99.6 | 100.0 | 0.579 | 0.45 |  | 96.9 | 98.5 | 1.012 | 0.31 |
| **Measles vaccination (%)** | 92.7 | 93.5 | 0.101 | 0.75 |  | 93.3 | 94.7 | 0.341 | 0.56 |
| **DPT vaccination (3 doses) (%)** | 93.9 | 93.5 | 0.036 | 0.85 |  | 89.5 | 88.3 | 0.154 | 0.70 |
| **Hepatitis B vaccination (3 doses) (%)** | 91.8 | 90.7 | 0.199 | 0.66 |  | 88.2 | 89.1 | 0.067 | 0.80 |
| **Polio vaccination (3 doses) (%)** | 95.0 | 94.2 | 0.120 | 0.73 |  | 92.1 | 91.4 | 0.066 | 0.80 |

**References**

1 Wang Y, Li X, Zhou M, et al. Under-5 mortality in 2851 Chinese counties, 1996-2012: a subnational assessment of achieving MDG 4 goals in China. Lancet 2016; 387: 273-83.

2 National Bureau of Statistics of the People's Republic of China. China Statistical Yearbook 2014. Beijing: National Bureau of Statistics of the People's Republic of China, 2014. http://www.stats.gov.cn/tjsj/ndsj/2014/indexch.htm (accessed May 25, 2017).
